# Drug Delivery Strategies for Age-Related Diseases

**DOI:** 10.3390/ijms25168693

**Published:** 2024-08-09

**Authors:** Kenichi Yoshihara, Michiko Horiguchi

**Affiliations:** Division of Pharmaceutics, Faculty of Pharmaceutical Sciences, Sanyo-Onoda City University, 1-1-1 Daigaku-Dori, Sanyo Onoda 756-0884, Japan

**Keywords:** drug delivery systems, age-related diseases, p16^INK4A^, SASP, senescence-associated β-galactosidase

## Abstract

Drug delivery systems (DDSs) enable the controlled release of drugs in the body. DDSs have attracted increasing attention for the treatment of various disorders, including cancer, inflammatory diseases, and age-related diseases. With recent advancements in our understanding of the molecular mechanisms of aging, new target molecules and drug delivery carriers for age-related diseases have been reported. In this review, we will summarize the recent research on DDSs for age-related diseases and identify DDS strategies in the treatment of age-related diseases.

## 1. Drug Delivery Systems for Age-Related Diseases

A drug delivery system (DDS) is a drug formulation technology that controls the pharmacokinetics of the active agent, enabling improved drug absorption, controlled drug release, and targeting of the site of action. DDSs aim to maximize therapeutic effects and reduce adverse reactions by delivering drugs to specific sites in the body at appropriate times and in correct doses. DDS products, such as spansules and other oral and transdermal controlled-release products, were developed in the 1950s, followed by the development of nanotechnology-based drug carriers. More recently, there have been major developments in drug carrier products, such as antibody–drug conjugates and nucleic acid drugs, that use nanotechnology [1,2]. The determination of disease-specific target molecules and formulation designs that enable targeting are important for the development of such DDSs. In this review, we focus on age-related diseases and summarize the major molecules used to target such diseases and the delivery strategies reported in the past 5 years (Table 1). We also outline the findings on target molecules and DDSs for age-related diseases. The methodology employed in the present literature review is delineated below. This review article was written in accordance with the guidelines set forth in the Scale for Assessment of Narrative Review Articles (SANRA) [3]. PubMed was the database used for the search. The inclusion criteria were as follows: articles published in the English language over a period of five years, from December 2018 to December 2023, and available as free full texts. The Boolean operator was used to retrieve search terms. Scientific articles containing the keywords “senescent cells”, “nanoparticles”, “senolytics”, “drug delivery system”, and “SASP” were included in the search. Only original papers were accepted; review papers were excluded. We searched 32,134 articles for “senescent cells”, 63,139 for “nanoparticles”, 893 for “senolytics”, 67,106 for “drug delivery system”, and 1161 for “SASP”. In addition, 309 articles were retrieved for “senescent cells” and “nanoparticles”, 6 for “SASP” and “nanoparticles”, and 3 for “nanoparticles” and “senolytics”. The following methodology was employed in the selection of papers for review: The database was searched using the specified keywords. Subsequently, the titles, abstracts, and publication dates of the retrieved articles were reviewed to identify those that were relevant to the purpose of this study. The most recently published studies were selected based on the date of publication. With regard to the article titles and abstracts, the most pertinent keywords were incorporated, and articles pertaining to “nanoparticles”, “prodrugs”, and “nucleic acid-based DDSs”, which are of significance in contemporary DDS research, were extracted.

## 2. DDS Target Molecules for Age-Related Diseases

Aging in individuals refers to the progressive dysfunction of multiple organs that occurs with age after reproductive maturity [12,13]. Age-related physiological dysfunction can lead to age-related diseases, such as cardiovascular diseases, neurodegenerative diseases, diabetes, and osteoporosis [13]. These disorders reduce the quality of life of patients and can worsen other diseases. Therefore, it is crucial to establish effective therapeutic agents for age-related diseases. Genomic instability, telomere shortening, epigenetic changes, disordered nutrient sensing, mitochondrial dysfunction, and stem cell depletion have been reported as mechanisms of aging [14]. Furthermore, the senescence process is suggested to involve cellular damage due to loss of protein and gene homeostasis, which induces cellular senescence and reduces damage to surrounding cells. Senescent cells are morphologically characterized by hypertrophy and flattening, irregular nuclear shapes associated with loss of the nuclear membrane component lamin B1, and cytoplasmic granulation [15,16,17]. The expressed biomarkers are characterized by high levels of senescence-associated lysosomal galactosidase activity (SA-β-Gal) and increased expression of cell cycle inhibitors (p21^WAF1/CIP1^, p16^INK4A^, p15^INK4B^, and p53). RT-qPCR, immunoblotting, and immunostaining methods can be used to confirm their expression levels [17]. In metabolism, senescent cells are also characterized by increased metabolic activity, including GSK3, AMPK, and mTOR pathways. [15,18,19]. Since no single marker characterizes senescent cells, it is recommended that senescent cells be identified using at least two senescence markers in addition to proliferation markers, such as Ki-67 antigen and bromodeoxyuridine [15]. The original papers reviewed in this paper utilized a variety of cancer cells, including SK-MEL-103 melanoma cells [7,20], NCI-H266 lung squamous cell carcinoma cells [20], MCF7 breast cancer cells [4], HFL-1 human lung fibroblast cells [5], A549 lung cancer cells [7,8], L1475(luc) lung adenocarcinoma cells [7], 4T1 breast cancer cells [7], HCT116 colon cancer cells [7], RAW264.7 cells [9], Huh7 cells [9], IMR90 cells [6,7], MLg cells [7], primary rat synovial cells [11], and HLECB3 cells [10], to induce cellular senescence. The accumulation of senescent cells exhibiting the aforementioned characteristics is associated with a number of age-related conditions, including Alzheimer’s disease, Parkinson’s disease, and other forms of neurodegeneration; atherosclerosis; type 2 diabetes; tissue fibrosis; cancer; gray hair; muscle loss; obesity; and glaucoma [17,21]. Among these age-related diseases, genome-wide association studies (GWASs) and candidate analyses have demonstrated that non-coding regulatory polymorphisms in the vicinity of the *CDKN2A* locus, which encodes p16^INK4A^, ARF, and p15^INK4B^, are responsible for the lifetime risk of melanoma, pancreatic cancer, leukemia, and glioma [21]. For example, these studies have shown that these polymorphisms are associated with an increased risk of developing these cancers. Additionally, studies have indicated a correlation between CDKN2A/B and other conditions, including arteriosclerotic diseases such as myocardial infarction, stroke, and aortic aneurysm, as well as type 2 diabetes, glaucoma, and endometriosis. Similarly, testing for non-malignant diseases with congenital changes in aging regulation has revealed that pulmonary fibrosis, which is targeted for treatment in this review and is a common feature of lung aging, involves TERT, a regulatory subunit of telomerase [21].

**Figure 1:**
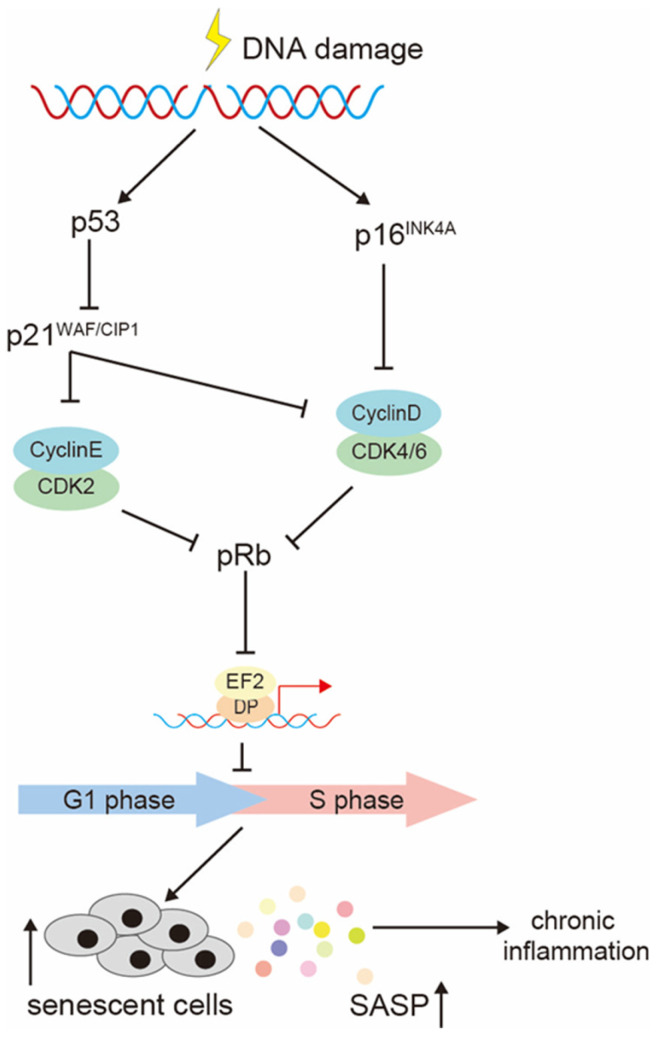
Mechanism of generation of senescent cells. One of the hallmarks of aging at the cellular level is a prolonged, generally irreversible cell cycle arrest accompanied by damage to secretory functions, macromolecules, and metabolic changes. DNA damage caused by age-related stress or physiological processes activates p53, which in turn activates the CDK2 inhibitor p21^WAF1/CIP1^ (CDKN1A). DNA damage also activates the CDK4/6 inhibitor p16^INK4A^ (CDKN2A). These factors dephosphorylate and persistently activate RB family proteins, resulting in inhibition of E2F transcriptional activation, cell cycle arrest, and accumulation of senescent cells.

Further, chronic cellular senescence disrupts intercellular communication and promotes aging [14]. The genes *p53* and *p16^INK4A^* are associated with the process of cellular senescence and represent important targets for the development of strategies aimed at suppressing this phenomenon. (Figure 1).

Furthermore, some studies have reported an association between senescence and inflammation [22,23]; the term “inflammaging” has been coined to describe this phenomenon [24].

**Figure 2:**
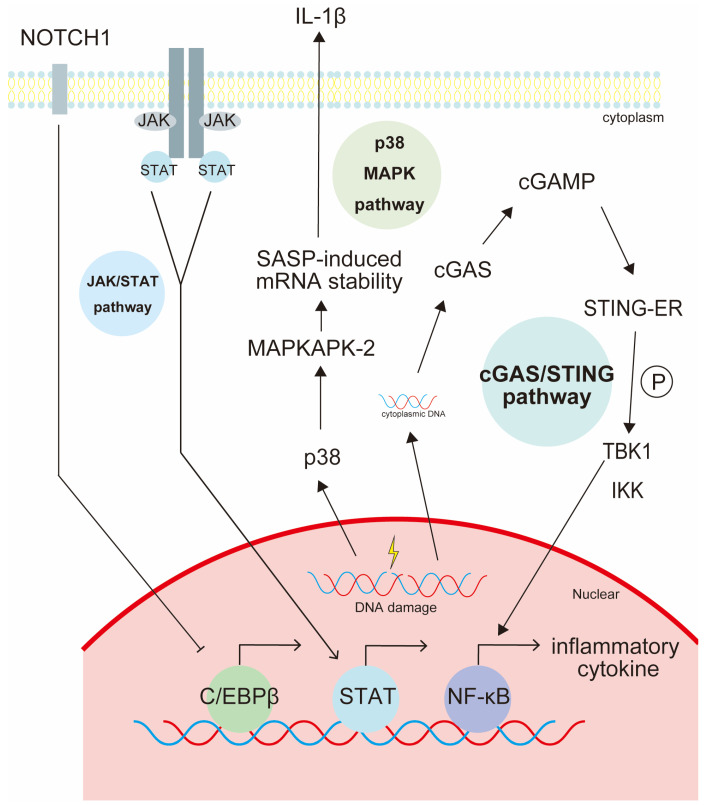
Cytoplasmic DNA from DNA damage in senescent cells can be an SASP-inducing signal. A typical signaling mechanism of SASP is the activation of the cGAS/STING pathway [26]. Typical transcriptional regulatory mechanisms of SASP are mainly regulated by two major factors, NF-κB and CCAAT/enhancer binding protein β (C/EBPβ). Inhibition of SASP by NOTCH1 results from repression of C/EBPβ transcription.

DNA damage to senescent cells induces a senescence-associated secretory phenotype (SASP) that is characterized by cytokine and protein secretion (Figure 2). Apoptosis of senescent cells in mice has been reported to delay senescence and tumorigenesis [25]. Currently, “senolytics” are being developed to induce senescent cell death [26]. Among the first senolytics identified were Dasatinib and Quercetin. Together, they have been shown to reduce the burden of senescent cells while improving healthy lifespan in several models, including aging and radiation-exposed mice [26]. SASP involves a complex network of signaling pathways that regulate the secretion of various factors by senescent cells. For example, as shown in Figure 2, the NF-κB pathway, the MAPK pathway, the JAK/STAT pathway, and the cGAS/STING pathway [27] are important signaling pathways that regulate SASP components [26]. The interaction of these signaling pathways regulates the expression and secretion of SASP components by senescent cells. Regulation of these pathways can modulate SASP-induced effects on tissue homeostasis, inflammation, and age-related diseases and could be targeted by senolytics. Furthermore, senescent cells retain β-galactosidase activity, lamin B1, p53, p21, and γH2AX, which have been used as biomarkers [8]. DDSs selective for senescent cells are being actively researched using these markers.

## 3. Nanocarriers Used as Senescent Cell-Targeting DDSs

**Figure 3:**
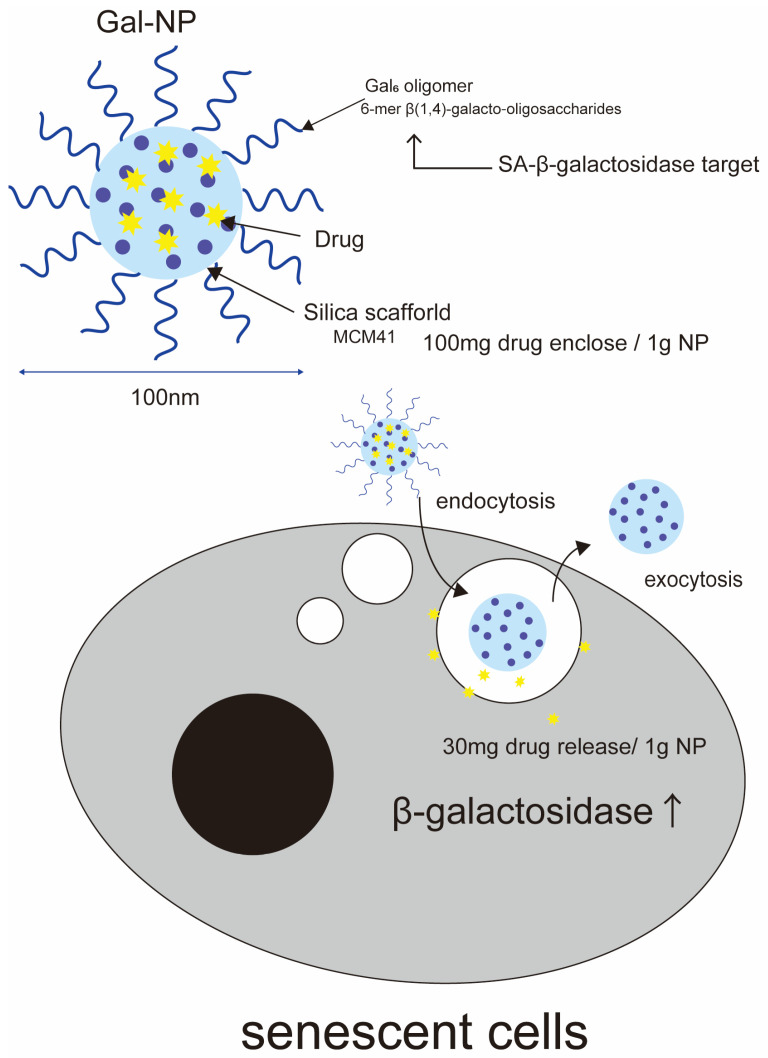
Silica-based scaffold (MCM41) capable of encapsulating a wide variety of drugs coated with a hexametric beta-1,4-galactooligosaccharide coating.

Senescence-associated β-galactosidase (SA-β-Gal) is the most widely used biomarker for senescent cells. SA-β-Gal is a lysosomal enzyme encoded by *GLB1* that specifically accumulates in senescent cells with elevated lysosomal contents [17,28]. In non-senescent cells, β-galactosidase is active at a pH of around 4.0. In senescent cells, SA-β-Gal activity is defined as β-galactosidase activity that is detectable at around pH 6.0. This makes Gal-NP beads versatile nanocarriers for senescent cell-targeted DDSs [20]. Gal-NP beads are based on a porous silica scaffold (MCM-41) encapsulated with a hexametric β-1,4-galactooligosaccharide coating and can encapsulate a variety of drugs (Figure 3). The drug delivery mechanism of this drug is as follows: a substantial proportion of senescent cells exhibit elevated levels of β-galactosidase activity. The targeted delivery of selected drugs to high levels of β-galactosidase in aging cells is achieved through the use of galactooligosaccharide-coated porous silica scaffolds. The system is degraded by the hydrolytic enzyme β-galactosidase, which is present in senescent cells, resulting in the release of the drug. The efficacy of this approach has been demonstrated in vitro in palbociclib-induced senescence with CDK6/CDK4 inhibitors in responding cancer cells and in vivo in palbociclib-induced senescence in tumor grafts and bleomycin-injured lungs, where preferential release of the encapsulated drug has been demonstrated. β-Gal digests the sugar coating of the beads and releases the drug from the silica scaffold. Generally, 100 mg of drug can be encapsulated in each gram of beads. Moreover, approximately 30 mg of drug is released per gram of beads when digested in vitro. Gal-NP beads have a particle size of approximately 100 nm; these can be taken up by senescent cells through endocytosis, and the bead portion is exocytosed after fusion with lysosomes.

The advantages of SA-β-Gal include its capacity to selectively identify senescent cells and its efficacy in addressing pulmonary fibrosis. The efficacy of GalNP(dox) was evaluated by encapsulating doxorubicin (GalNP(dox)), which has the ability to kill both senescent and non-senescent cells and exhibits intrinsic fluorescence. In contrast, exposure to free doxorubicin resulted in the induction of senescence, regardless of the presence of the drug, thereby demonstrating the selectivity of GalNP(dox) for senescent cells and its efficacy. In vivo, the administration of bleomycin-induced pulmonary fibrosis was significantly improved in comparison to free doxorubicin administration. A further limitation of DDS formulations utilizing SA-β-Gal is that β-galactosidase activity is not an entirely accurate marker of senescent cells. The incomplete targeting is corroborated by the observation that silicon levels quantified by ICP-MS (inductively coupled plasma mass spectrometry) in the lungs of mice with bleomycin-induced pulmonary fibrosis exhibited comparable levels in both healthy and fibrotic lungs. β-galactosidase activity is not solely confined to aging cells. In addition to the evidence indicating that elevated levels have been documented in damaged and diseased tissues as well as in aging cells, the drug reaches tissues irrespective of whether they are healthy or diseased. This is particularly relevant for elderly patients with age-related diseases, who are prone to presenting with multiple comorbidities. In the event that targeting is incomplete and the patient is concurrently afflicted by additional maladies, the release of the pharmaceutical agent may occur at sites other than the intended target, where β-galactosidase activity is elevated. This may result in the emergence of unintended off-target effects.

The following validations and issues may be considered to ensure clinical safety and validity for the clinical application of DDS formulations utilizing SA-β-Gal. It has been demonstrated that β-galactosidase activity is present at elevated levels in damaged and diseased tissues, as well as in aging cells. Moreover, the drug is capable of reaching tissues irrespective of their physiological state. This indicates that the targeting is not optimal and may result in unintended off-target effects. In particular, in the context of age-related diseases, it is possible that other diseases may be present in addition to the specific disease under consideration. Therefore, while this paper evaluated the reduction in side effects of platelet suppression and cardiac hypertrophy, further in vivo evaluation of side effects in the presence of other diseases in combination is recommended. Furthermore, as previously stated in the paper, drug release was observed in pulmonary macrophages in both the control and aged cell groups. The evidence that macrophages are capable of metabolizing nanoparticles also suggests that the targeting is not entirely precise. Accordingly, additional assessment of the impact of nanoparticles on the macrophage population, the influence on immunity, and the effect of formulation attributes, including the potential for particle size to be engulfed by macrophages, is essential to guarantee further clinical safety. It is necessary to evaluate the temporal control of the drug in vivo, as no information on pharmacokinetics was provided. In addition to the in vivo evaluation described above, it is also necessary to conduct a formulation stability evaluation and establish a stable storage method in order to facilitate the use of the product in actual clinical practice.

**Figure 4:**
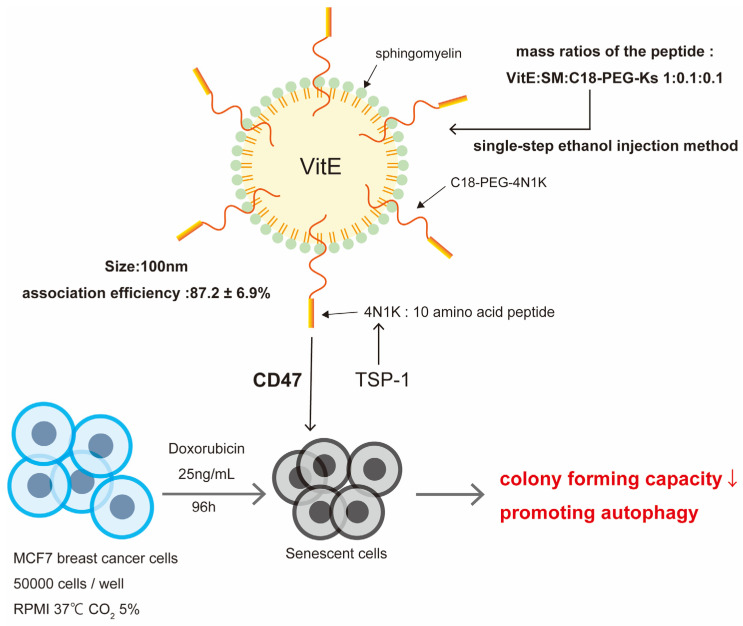
The nanosystem, comprising sphingomyelin and coated with 4N1K, is designed to target CD47 receptors. TSP-1/CD47 receptors exert an inhibitory effect on the replication and proliferation of cells.

The 4N1K–sphingomyelin nanosystem [4] targets the CD47 receptor of senescent and cancer cells to inhibit cell replication and proliferation. It acts by modifying the nanoparticle surface with a 4N1K peptide comprising C-terminal 10 amino acids from TSP-1 (Figure 4). The mechanism of action of the 4N1K–sphingomyelin nanosystem is the inhibition of self-renewal and cell proliferation by the homotrimeric glycoprotein TSP-1/CD47 receptor. It has been shown to prevent chemotherapy-induced breast and colon cancer cells from evading senescent cells [29]. CD47 expression is regulated by p21^WAF1^. The downregulation of p21^WAF1^ by cell cycle inhibitors, in turn, upregulates *Myc*, which binds to the CD47 promoter and suppresses CD47 expression, thereby avoiding senescence. Derivatives of the C-terminal domain of TSP-1, designated 4N1Ks, have been demonstrated to stimulate autophagy in senescent and cancerous cells, while simultaneously impeding their evasion from senescent cells. We have targeted CD47 using nanoparticles modified with C18-PEG-Ks, in which the 4N1Ks are connected to PEG by a carbon chain (C18). The nanoparticles to be coated exhibit good biodegradability and therapeutic compatibility with sphingomyelin, which is present in cell membranes, and vitamin E, which is commonly used for encapsulation of various therapeutic molecules. The efficacy of DDSs in this paper is demonstrated in in vitro experiments on MCF7 breast cancer cells, with targeting to CD47 by 4N1Ks derived from the C-terminal domain of TSP-1 against chemically induced senescent cells using doxorubicin. In doxorubicin senescence-induced MCF7 breast cancer cells, a peptide dose of 0.5 μM has been shown to significantly reduce the number of cancer cell colonies compared to untreated and 4N1K blank groups. This system is relatively easy to prepare, involving a one-step ethanol injection method, and exhibits excellent colloidal properties, low cytotoxicity, and high cell internalization. Process reproducibility and scalability are enhanced by this efficient and simple ethanol injection method. The use of biodegradable and biocompatible components, such as sphingomyelin and vitamin E, in SN formulations ensures system safety and stability for drug delivery applications. In terms of physicochemical properties, the average particle size is reported to be approximately 108 nm, and the SNs-Ks association efficiency (the percentage of peptides associated with the nanosystem) is 87.2 ± 6.9%. These physicochemical properties contribute to the efficacy of nanosystems for the targeted delivery of drugs to aging cells.

One advantage of the 4N1K–sphingomyelin nanosystem is that the incorporation of the 4N1K peptide into the nanosystem successfully reduces the effective concentration to 1/10, thereby reducing the dosage. Furthermore, evidence indicates that CD47, which is involved in the inhibition of efferocytosis, is overexpressed not only in cancer cells but also in atherosclerotic lesions [30]. This results in the accumulation of apoptotic cells and the promotion of atherosclerosis. In atherosclerotic plaque, CD47 expression is upregulated by TNF-α. Consequently, CD47-targeted DDS formulations can be employed not only in anti-cancer therapy but also in the treatment of atherosclerosis. Conversely, the 4N1K–sphingomyelin nanosystem is constrained by limitations pertaining to its targeting capacity and physical properties. CD47, the target of the DDS in this study, is universally present, and its capacity to target senescent cells is believed to be limited. The physical properties of the formulation in RPMI medium with 1% FBS exhibited a particle size of 272 ± 10 nm and a zeta potential of −11 ± 1 mV. While an in vitro evaluation was conducted in this study, an in vivo evaluation was not. Accordingly, the actual kinetics in the bloodstream remain unknown. However, given the particle size, it is plausible that the drug is taken up by phagocytes, such as macrophages. Additionally, the zeta potential was diminished from +59 ± 9 mV in the absence of 1% FBS supplementation due to the adsorption of plasma proteins, thereby enhancing colloidal stability. Nevertheless, the outcome of −11 ± 1 mV, even following a reduction in the zeta potential, indicates the potential for aggregation of the formulation, which has been a persistent challenge. It is essential to assess the impact of this aggregation on in vivo pharmacokinetics.

It is essential to consider the following validations and issues to guarantee the clinical safety and suitability of DDS formulations that employ the 4N1K–sphingomyelin nanosystem for clinical use. An in vivo evaluation is required. CD47 is an incomplete target for senescent cells and may exhibit toxicity in unexpected tissues and organs. It is therefore essential to evaluate the side effects of the drug in vivo, with particular attention paid to those related to the immune system. Toxicity and dose design must also be evaluated based on dosage. In addition, pharmacokinetics must be evaluated, as must the effects of the drug’s physical properties. Moreover, the DDSs utilized in this study are PEGylated lipid nanoparticles. Following administration, lipid nanoparticles may encounter barriers such as localization in unexpected tissues due to adsorption of plasma proteins, predation by phagocytes, and disintegration of the formulation in the bloodstream due to the effects of complement and other substances. Furthermore, it is important to note that lipid nanoparticles are often metabolized in the liver. Therefore, it is essential to conduct a comprehensive evaluation of their pharmacokinetics in this organ. In the absence of data regarding pharmacokinetics, it is essential to assess the temporal control of the drug in vivo. In addition to the in vivo evaluation described above, it is also necessary to conduct a formulation stability evaluation and establish a stable storage method in order to facilitate the use of the drug in actual clinical practice.

## 4. Nucleic Acid-Based DDSs Targeting Senescent Cells

**Figure 5:**
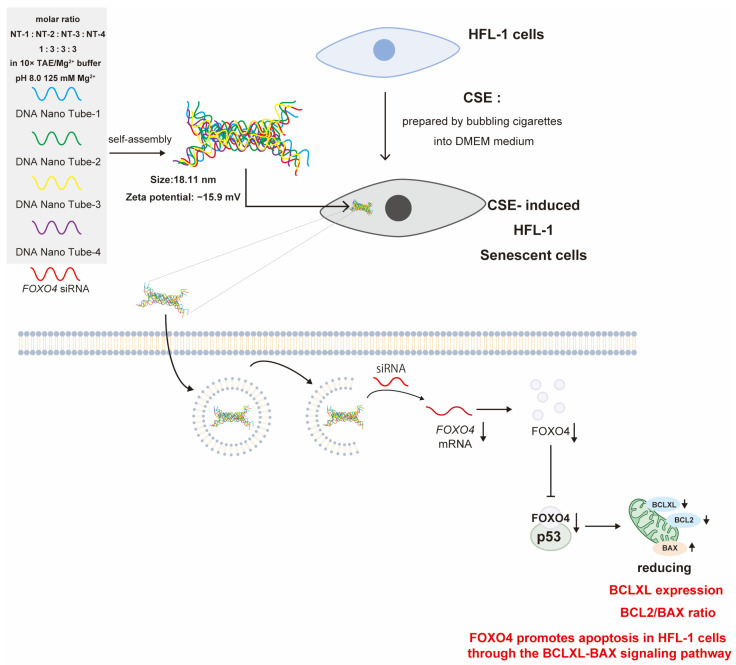
The drug increases BCLXL expression in CSE (cigarette smoke extract)-induced aged HFL-1 cells, decreases the BCL2/BIX ratio, and selectively eliminates HFL-1 cell activity.

Apoptosis can be induced by inhibiting the direct interaction between p53, which is important for DNA repair, cell growth arrest, and apoptosis, and FOXO4, a transcription factor [31]. The objective of the DDS formulation in the latter study was to achieve FOXO4 knockdown, which has been demonstrated to be elevated in both in vitro and in vivo models of cigarette smoke extract (CSE)-induced aging lung fibroblasts (HFL-1 cells). *FOXO4* siRNA encapsulated in DNA nanotubes (DNA-NTs) was delivered to the lungs, resulting in a concentration- and time-dependent reduction in FOXO4 levels in CSE-induced senescent HFL-1 cells. This effect was selective for CSE-induced senescent HFL-1 cells. The efficacy of si*FOXO4* -NTs was demonstrated in vitro, where they were incorporated into CSE-induced senescent HFL-1 cells in a dose-dependent manner in Cy5-incorporated cellular uptake experiments. Furthermore, evaluation by calcein/PI staining demonstrated that CSE-induced senescent HFL-1 cells were selectively eliminated in a dose- and time-dependent manner, with no discernible impact on CSE-untreated HFL-1 cells. si*FOXO4* -NTs have been reported as DDSs that silence FOXO4 induced by tobacco smoke with siRNA and reduce and eliminate increased BCLXL and BCL2/BAX ratios in SCE-induced senescent HFL-1 cells [5] (Figure 5).

The si*FOXO4* -NT formulation offers the advantage of reducing FOXO4 expression without affecting normal HFL-1 cells. Furthermore, it has been demonstrated to promote apoptosis in CSE-induced aged HFL-1 cells. CSE-induced aged HFL-1 cells that had not been treated with CSE exhibited a significant reduction in BCLXL expression and BCL2/BAX ratios following treatment with *FOXO4* siRNA and si*FOXO4*-NTs. In contrast, the effects of *FOXO4* siRNA and si*FOXO4*-NTs on the BCL2/BAX ratio were found to be insignificant in CSE-untreated HFL-1 cells. A further limitation of si*FOXO4*-NTs is that DNA nanotubes (DNA-NTs) may have low in vivo stability. Pharmaceuticals utilizing nucleic acids are subject to the following limitations: degradation by nucleases within the body; induction of immune function due to recognition as foreign molecules by TLRs; high molecular weights and large sizes; polyanionicity and high polarity due to the negative charge of the phosphate groups; and hydrophilicity, which impedes their ability to penetrate cell membranes and demonstrate efficacy [32,33]. The hydrophilic nature of nucleic acids hinders their ability to permeate cell membranes, thereby limiting their effectiveness. Moreover, despite the low in vivo stability, pharmacokinetic evaluation is imperative to ascertain the potential long-term effects of genetic modification. In terms of formulation characteristics, the zeta potential is −15.9 mV, which indicates a possibility of aggregation. Therefore, an evaluation of formulation stability is also necessary.

The following validations and issues may be considered to ensure the clinical safety and appropriateness of si*FOXO4*-NT-based DDS formulations for clinical application. It has been demonstrated that the removal of aged pulmonary endothelial cells by intraperitoneal injection or orally with FOXO4-DRI (D-Retro-Inverso) has a deleterious effect on pulmonary hemodynamics in a mouse model of pulmonary hypertension. Direct delivery to the lungs is therefore required, as has been confirmed. Nevertheless, a significant challenge remains: the lack of consideration of specific delivery methods in the existing literature. It would be beneficial to consider methods of direct delivery to the lungs, such as nebulization, which could be employed in actual clinical practice. In this instance, it is crucial to ascertain the stability of the formulation and the particle size for optimal alveolar delivery. In the case of intravenous delivery, it is necessary to consider modifying the DNA-NTs in order to enable actual pulmonary delivery, or to encapsulate the DNA-NTs in cationic lipid particles if the particles are not stable. Furthermore, in light of the potential for distribution to unanticipated tissues due to protein binding and other factors, an in vivo pharmacokinetic evaluation of the sort previously described is recommended. Furthermore, as the product is a DNA-based entity, it is imperative to assess its potential immunogenicity and the associated risk to genome integrity. In the absence of data pertaining to pharmacokinetics, an in vivo assessment of the temporal regulation of the drug is imperative. In addition to the in vivo evaluation described above, it is also necessary to conduct a formulation stability evaluation and establish a stable storage method for DNA-NTs in order to facilitate their use in actual clinical practice.

**Figure 6:**
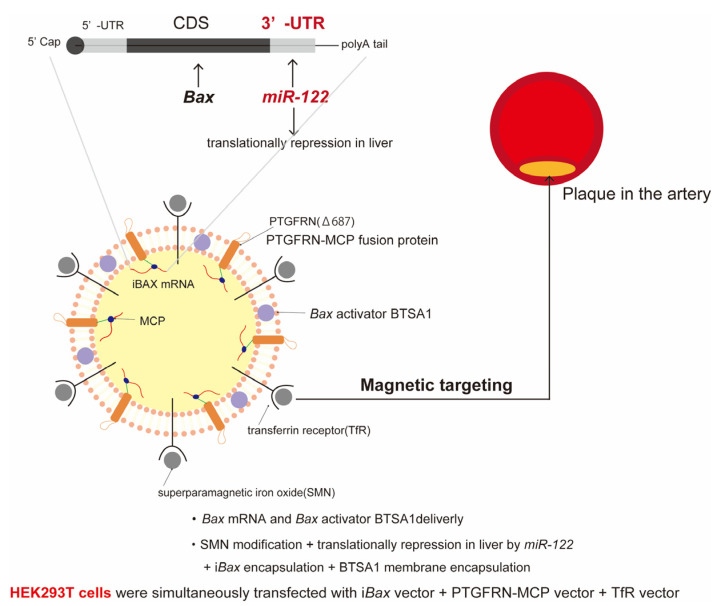
Magnetic nanoparticle SMN + *miRNA-122* liver translational repressor + i*Bax* mRNA (BAX; BCLA-2-related protein X) encapsulation + BTSA1 (BAX activator) membrane loading + EV (extracellular vesicle).

To prepare si*FOXO4*-NTs, computer programs such as SEQUIN were used to design four unique DNA strands that are mixed in a specific molar ratio and self-assemble into a nanotube structure. In summary, this study shows that self-assembling DNA nanotubes loaded with *FOXO4* siRNA can selectively and effectively reduce FOXO4 levels in senescent fibroblasts, suggesting a new approach for COPD treatment. 

i*Bax* mRNA/BTSA1 delivery by magnetic extracellular vesicles [9] has also been reported as a senescent cell-targeting DDS to treat atherosclerotic plaques (Figure 6). BCL-2-associated protein X (BAX) is a pro-apoptotic protein. This DDS is an extracellular vesicle (EV) that targets *Bax* mRNA (i*Bax*) and a *Bax* activator (BTSA1) for delivery to lesions and inflammation sites using superparamagnetic iron oxide nanoparticles (SMNs). The mechanism of EV^iTx^ treatment is based on the delivery of i*Bax* mRNA/BTSA1 to senescent cells, which have been observed to have a significantly lower anti-apoptotic reserve. The study employed an “activate the activator” strategy, whereby *Bax* mRNA and the activator BTSA1 were delivered directly to senescent cells. The objective was to induce the elimination of senescent cells through the promotion of apoptosis. Furthermore, extracellular vesicles (EVs) are highly effective vehicles for the delivery of nucleic acids due to their low immunogenicity and high biocompatibility. However, they tend to accumulate in the liver and have been observed to exhibit hepatotoxicity. To circumvent this issue, a modified *Bax* mRNA with a *miR-122-5p* (*miR-122*) recognition site in the 3′-UTR region was employed to preclude translation between the two and thereby mitigate the incidence of side effects. This offers a means of reducing the adverse effects of any unintended delivery that occurs outside of the liver. The targeting of senescent cells in atherosclerotic plaques presents a significant challenge due to the heterogeneity of these cells and the lack of a consistent origin. To overcome this challenge, superparamagnetic iron oxide nanoparticles (SMNs) were integrated into the system to facilitate EV delivery irrespective of cell type and contingent on spatial recognition. In vitro, simultaneous transfection of *Bax* mRNA and activator BTSA1 in EV (EV^iTX^) induced apoptosis in aged foamy macrophages but showed no significant effect on non-aged macrophage hepatocytes (Huh7 cells). These findings indicate that EV^iTx^ is a safe senescent cell removal strategy that preferentially kills senescent cells.

The advantage of i*Bax* mRNA/BTSA1 delivery by magnetic extracellular vesicles is that the efficacy of EV^iTx^ in delivering *Bax* mRNA to plaque sites through repeated administration to the tail veins of ApoE^−/−^ mice has been demonstrated. Furthermore, treatment with EV^iTx^ has been shown to result in a notable reduction in atherosclerotic plaque. This was particularly evident in the lipid core in comparison to the control group. In contrast, the limitations of i*Bax* mRNA/BTSA1 delivery by magnetic extracellular vesicles, as outlined in the paper, include pulmonary and splenic complications resulting from i*Bax* mRNA accumulation, augmented apoptosis levels with EV^iTx^ treatment, and EV^iTx^’s potential as a promising alternative EV source for clinical applications. Given the ease with which HEK293T cells can be modified, EVs derived from these cells have been the delivery medium of choice. Nevertheless, the oncogenic nature of HEK293T cells renders them unsuitable as a delivery platform, thereby imposing constraints on the applicability of engineered EVs.

The following validation and issues should be considered to ensure the clinical safety and validity of DDS formulations using i*Bax* mRNA/BTSA1 delivery EV^iTx^ therapy for clinical application. First, the side effects observed in the lung and spleen must be re-evaluated in vivo by modifying the *Bax* mRNA to avoid translation in each organ, as was performed with *miR-122* for hepatotoxicity. Mesenchymal stem cells, such as ADSCs, represent a promising alternative source of EVs for the cancer origin of HEK293T cells. However, reproducibility, including the difficulty of genetic modification, will be a significant challenge. Furthermore, stability and pharmacokinetic evaluations of this formulation have yet to be conducted. In order to transition to clinical practice, it is essential to assess the formulation’s stability, as the storage method must be considered. The formulation is prepared through a complex process. Furthermore, the financial implications of this modification and the reproducibility of the formulation must be assessed.

This study suggests that eliminating senescent cells and reducing SASP factors may be beneficial for age-related diseases other than atherosclerosis, such as chronic kidney disease, Alzheimer’s disease, Parkinson’s disease, and cancer, which are associated with senescent cells and chronic inflammation.

## 5. Galactose-Modified Prodrugs

**Figure 7:**
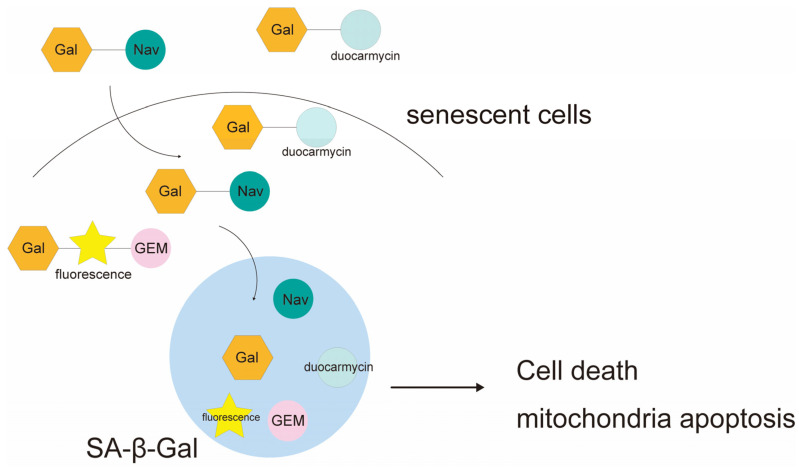
In a β-galactosidase-dependent manner, these agents induce apoptosis of senescent cells.

Increased lysosomal and galactosidase activity is observed in senescent cells. Therefore, prodrugs have been developed that release activated drugs in senescent cells by hydrolyzing galactose. The following studies have also shown that the combination of galactose with drugs can increase the safety of the drugs and add non-therapeutic uses. Actual galactose-modified prodrugs include galactose-modified duocarmycin (GMD) [6], galactose-bound Navitoclax (Nav-Gal) [7], and a targeted supramolecular prodrug (TSPD) designed to destroy senescent cells for chronic renal failure [8] (Figure 7).

GMD [6] is a galactose-modified prodrug of duocarmycin, a potent inhibitor of cell growth. It is preferentially processed in senescent cells and induces apoptosis of senescent cells in a β-galactosidase-dependent manner. When treated with β-Galactosidase, the GMD prodrug is converted to an active duocalmycin derivative that is β-galactosidase-dependent and selectively cytotoxic to senescent cells. GMD prodrugs have a bystander effect that allows them to selectively kill senescent cells at low concentrations without having an effect on normal cells. With regard to the efficacy of GMD, in vivo studies have demonstrated that GMD administration for four consecutive days, two months after whole-body radiation therapy (6 Gy), resulted in the significant elimination of radiation-induced senescent cells. This was evidenced by a reduction in SA-β-Gal activity, CDKN1A encoding p21^CIP1^, SASP component IL-6, and Cxcl1 expression.

The advantage of galactose-modified duocarmycin (GMD) prodrugs is that they can also be employed to eradicate precancerous senescent cells. The *Hesx1^Cre/+^*; *Ctnnb1^lox(ex3)/+^* ACP mouse model demonstrated that GMD prodrugs can eliminate tumorigenic senescent clusters. An in vivo evaluation of whether treatment with GMD could eliminate β-catenin-accumulating senescent cell clusters preferentially without affecting other cell types in the pituitary, such as synaptophysin+ cells, indicated that it could. Conversely, GMD has a limitation in that its effect is contingent upon SA-β-Gal activity. This is evidenced by the fact that in vitro experiments demonstrated that 2.5 μM GMD treatment resulted in the death of the majority of senescent cells, while some senescent cells with low SA-β-Gal activity survived. The aforementioned results indicate that the efficacy of the drug is contingent upon SA-β-Gal activity, thereby rendering it challenging to determine the optimal dosage and assess the pharmacokinetics.

It is essential to consider the following validations and issues to guarantee the clinical safety and appropriateness of galactose-modified duocarmycin (GMD) prodrug formulations for clinical application. Given that the efficacy of GMD is contingent upon SA-β-Gal activity and that the safety of the drug is also influenced by this activity, it is imperative to develop a dosage regimen that is both efficacious and safe to administer in a clinical setting. Accordingly, a dosage regimen must be designed that can be safely employed in a clinical setting for each disease. Should the aforementioned dosage design become viable, this formulation could be employed in conjunction with cisplatin, potentially forming the basis of novel cancer regimens. Furthermore, as no pharmacokinetic evaluation has been conducted in the existing literature, it is necessary to evaluate the impact of prodrug conversion on the drug’s half-life and tissue localization in vivo. Moreover, the stability of the formulation must be evaluated, as the storage method for the formulation must be taken into consideration prior to the transfer of the drug to actual clinical use. In summary, GMD prodrugs are designed to utilize the characteristic SA-β-Gal activity of senescent cells for targeting. The conversion of GMD to active duocalmycin in senescent cells has been demonstrated to induce cell death through processes such as DNA alkylation. This drug may alleviate the states of diseases associated with the aging process, such as cancer and myofibrosis. By ameliorating senescent cell-mediated inflammation and tissue dysfunction as described above, this prodrug may improve outcomes and quality of life in aging populations.

Nav-Gal [7] is a conjugated drug composed of Navitoclax and galactose that targets β-galactosidase. The detailed mechanism of action of the galactose-linked derivative of navitoclax (Nav-Gal) is as follows: Initially, the compound is taken up passively by both aging and non-aging cells. In non-aging cells, the compound becomes inactive by binding to degradable galactose, thereby preventing its ability to inhibit anti-apoptotic proteins such as BCL-2 and induce apoptosis. In contrast, in senescent cells, augmented lysosomal and galactosidase activity—a defining feature of cellular senescence—facilitates the hydrolysis of galactose and the subsequent release of activated Navitoclax into the cytoplasm of senescent cells. The anti-apoptotic BCL-2 protein, which is overexpressed in senescent cells, is inhibited by free Navitoclax, thereby promoting the specific apoptosis of these cells. This is a consequence of the hydrolysis of galactopyranosides that are covalently attached to the N of bis(sulfonyl)aniline of Navitoclax, which occurs in the presence of β-galactosidase. The efficacy of Nav-Gal was demonstrated by parallel treatment of cisplatin-induced senescent cells with Navitoclax or Nav-Gal in vitro. Real-time measurements of apoptosis revealed that Nav-Gal induced apoptosis and killed senescent cells to a greater degree than Navitoclax and prevented non-apoptosis. Previous studies have shown that Nav-Gal prevents senescent cell death to a greater degree than Navitoclax. Furthermore, the clonogenicity assay demonstrated that the combination of cisplatin and Navitoclax, Nav-Gal, and the control exhibited significantly greater inhibitory effects on cell growth compared to monotherapy. This was observed in the context of senescence-inducing cisplatin. The in vivo efficacy of the treatment was evaluated using a model in which A549 cells were subcutaneously implanted in the flanks of severe combined immunodeficient (SCID) mice. The combination of cisplatin with either navitoclax or nav-gal demonstrated comparable efficacy in inhibiting tumor growth. However, when administered without cisplatin, these agents exhibited no discernible impact on tumor growth. This finding indicated that the simultaneous induction of senescence by cisplatin was a prerequisite for the therapeutic activity observed. Moreover, when both Navitoclax and Nav-Gal were administered consecutively one week after cisplatin administration, a therapeutic effect on tumor growth was observed, indicating the potential for its use as an adjunctive therapy to eliminate senescent cells.

The most significant advantage of the galactose-linked derivative of Navitoclax (Nav-Gal) is its ability to generate a Nav-Gal prodrug that mitigates Navitoclax-induced thrombocytopenia at concentrations that align with physiological norms. In vivo, therapeutic doses of Nav-Gal were administered to evaluate the protection of platelet levels in wild-type C57BL/6J mice treated with the drug for a total of 10 days. The results demonstrated that thrombocytopenia did not occur in the Nav-Gal-treated group, and platelet counts were comparable to the control treatment group, indicating a reduction in platelet toxicity compared to Navitoclax. The platelet counts were found to be comparable to those of the control group, indicating a reduction in platelet toxicity relative to Navitoclax. Conversely, a limitation of Nav-Gal is that its efficacy is contingent upon SA-β-Gal activity, and its therapeutic activity necessitates the concurrent induction of senescence by cisplatin. The in vivo efficacy of the compound was evaluated using A549 cells that had been subcutaneously transplanted into SCID mice. When administered without cisplatin, the drug demonstrated no discernible impact on tumor growth, indicating that concurrent induction of senescence with cisplatin is a prerequisite for therapeutic efficacy. Cisplatin is nephrotoxic and, as a result, cannot be used in all cases, including those of patients with impaired renal function. In such cases, dose adjustments are required, particularly in elderly patients with impaired renal function. The aforementioned factors may potentially diminish the efficacy of cisplatin.

The following validations and issues should be considered to ensure the clinical safety and validity of the Navitoclax galactose-linked derivative (Nav-Gal) formulations for clinical application: The therapeutic activity of Nav-Gal in the treatment of cancer requires the simultaneous induction of senescence by cisplatin. Nav-Gal is required for the simultaneous induction of aging by cisplatin for the therapeutic activity of Nav-Gal. Therefore, an in vivo evaluation of renal, hepatic, and cardiac functions during simultaneous administration of cisplatin, which may cause nephrotoxicity, is necessary. Furthermore, a therapeutic dose of Nav-Gal must be designed in accordance with the adjusted dose of cisplatin for each renal function in order to facilitate further clinical application. Should the aforementioned dosage design become viable, this formulation could be employed in conjunction with cisplatin, potentially forming the basis of novel cancer regimens. Moreover, as the effects of prodrugs on the half-life and tissue localization of drugs have yet to be demonstrated in the literature, in vivo evaluation is imperative. Moreover, the stability of the formulation must be evaluated, as the storage method for the formulation must be considered prior to the transfer of the drug to actual clinical use. 

TSPD [8] is a prodrug formed by binding gemcitabine to the galactose moiety activated by SA-β-Gal. It is activated by SA-β-galactosidase and has been reported to improve renal function in mice with chronic renal failure (CRF). The detailed mechanism of action of TSPD is as follows: TSPD contains three functional parts. The first is a fluorescent carrier with a coumarin backbone. The second is a β-galactoside bond in the backbone that is selectively induced by SA-β-gal. The third is gemcitabine, a cytotoxic agent. TSPD is selectively activated by SA-β-gal, thereby enabling the sequential release of both a fluorophore for fluorescent imaging and free gemcitabine for chemotherapy. This initiates an autoimmune process that releases the parent drug, gemcitabine, which then reacts with surrounding nucleophiles to release an active methylenebenzopyrandione (MBP), thereby producing a fluorescent signal. In vitro experiments have demonstrated that the quantity of gemcitabine released is dependent on the concentration of SA-β-gal. The efficacy of TSPD has been evaluated through the use of fluorescence imaging and in vitro assays of selective cell death in cells that overexpress β-galactosidase. TSPD has been demonstrated to be selectively activated in senescent cells, releasing fluorophores and gemcitabine, and may serve as an effective senescent cell-depleting agent in the treatment of age-related diseases. The efficacy of TSPD was evaluated using NRK-52E cells, a type of rat renal tubular epithelial cell relevant to the treatment of CRF. Following culture with TSPD, no significant fluorescent signal was observed in non-aged cells. However, fluorescence was significantly enhanced in aged NRK-52E cells, confirming that TSPD targets aged renal cells. Therefore, it can be concluded that TSPD targets senescent kidney cells. Furthermore, cell viability testing of TSPD was conducted using the CCK-8 assay, which demonstrated that even concentrations as low as 0.01 μM were capable of selectively eliminating senescent NRK-52E cells.

A further advantage of TSPD is that it also inhibits renal fibrosis. The unilateral renal ischemia–reperfusion injury (UIRI) model was used to examine the effects of TSPD on renally impaired mice. The mice were treated with either a control, gemcitabine, or TSPD via the intraperitoneal injection (twice weekly for 3 weeks) route of administration. After 21 days, blood urea nitrogen (BUN) and serum creatinine (SCr) levels were measured to assess renal function in each group of mice. The results were evaluated. The results demonstrated that both BUN and SCr levels decreased to within the normal range after treatment with TSPD. Furthermore, the kidneys of the control group exhibited notable atrophy and decreased in weight compared to the kidneys of the TSPD (5 mg/kg)-treated group, and significant improvement was observed after treatment. A limitation of TSPD is that its effect is dependent on SA-β-Gal activity. It has been demonstrated that the effect of Nav-Gal is dependent on SA-β-Gal activity and that simultaneous induction of senescence with cisplatin is necessary for the therapeutic activity of Nav-Gal. TSPD may also be capable of enhancing its efficacy by inducing cellular senescence in combination with other agents. Moreover, as with GMD, the efficacy of TSPD is contingent upon SA-β-Gal activity, rendering dosage design and pharmacokinetic evaluation a challenging endeavor.

The following validation and issues should be considered to ensure the clinical safety and validity of TSPD prodrug formulations for clinical application. The literature provides detailed descriptions of the mechanism of action and efficacy evaluation of TSPD prodrug formulations, both in vitro and in vivo. However, there is a paucity of information regarding the evaluation of potential side effects. Gemcitabine has been employed for TSPD, and the occurrence of adverse effects, including myelosuppression, interstitial pneumonia, and renal dysfunction, cannot be ruled out. Although no toxicity was observed at doses as high as 25 mg/kg over a 2-week period, it would be prudent to evaluate toxicity over a longer period of time after use. Furthermore, since the pharmacokinetics, including the impact on the drug’s half-life and tissue distribution, were not demonstrated, an in vivo evaluation is deemed essential. Moreover, the stability of the formulation must be evaluated, as the storage method used for the formulation must be considered prior to the transfer of the drug to actual clinical use.

Galactose-modified prodrugs are versatile and can be applied to various drugs, and their efficacy and safety in various age-related diseases are expected to be verified.

## 6. Nanoparticle DDSs for Metallic Materials

**Figure 8:**
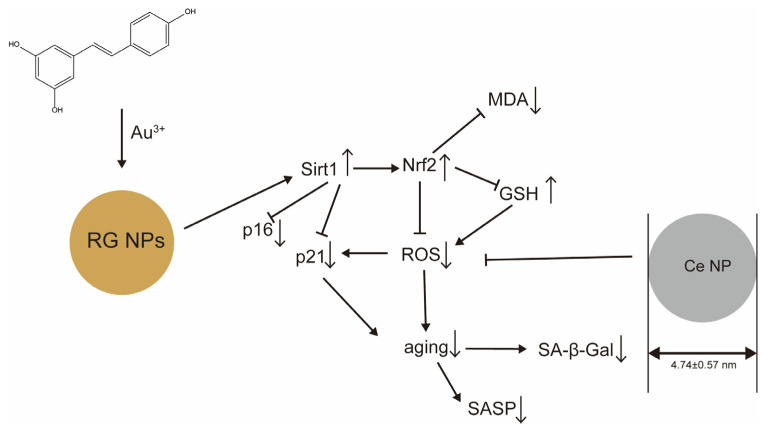
Resveratrol-encapsulated gold nanoparticles (RGNPs) inhibit cellular senescence and suppress SA-β-Gal and SASP, resulting in anti-aging and anti-inflammatory effects and reducing cataract symptoms. Ceria nanoparticles (CeNPs) inhibit osteoarthritis by suppressing not only ROS but also p16, p21, iNOS, COX2, MMP3, ADAMTS5, IL-6, and TNFα.

Ceria nanoparticles (CeNPs) [10] are formulations that inhibit osteoarthritis. Cerium is a lanthanoid metal element that exists as a mixture of trivalent and tetravalent ions. The conversion of cerium ions between trivalent and tetravalent states enables the repeated reduction of cerium. The majority of oxygen vacancies and Ce^3^⁺ are located on the surface. Consequently, CeNPs with a higher surface area-to-volume ratio exhibit greater reducibility than cerium oxide, which has larger particles. Synthesized cerium nanoparticles (CeNPs) exhibited good stability and a robust ROS scavenging effect (Figure 8), with an average size of 4.74 ± 0.59 nm. The in vitro efficacy of CeNPs in MP- and H_2_O_2_-induced aging synoviocytes demonstrated an increase in ROS concentration and the percentage of SA-β-Gal-positive cells, as well as an upward regulation of SASP-related biomarkers, including iNOS, COX2, MMP3, ADAMTS5, IL-6, and TNFα. The concentration of ROS and the percentage of SA-β-Gal-positive cells were found to decrease following treatment with CeNPs, with a concomitant suppression of SASP-related biomarkers. Furthermore, CeNPs suppressed the protein levels of p-p65 and p-IκBα in synovial cells cultured in multiple passages, attenuated IκBα degradation, and decreased p-p65 expression in OA rats following CeNP injection. This resulted in the inhibition of NFκB pathway activity in aging synovial cells via ROS removal. It was demonstrated that SASP can be inhibited.

Regarding the advantages of CeNPs, it was demonstrated in both in vitro and in vivo studies that not only ROS levels but also levels of p16, p21, iNOS, COX2, MMP3, ADAMTS5, IL-6, and TNFα were suppressed in the synovium. These reductions occurred following intra-articular injection. A limitation of the study was that the concentration of SASP protein in the joints could not be quantified due to the unavailability of sufficient rat joint fluid. A necessary control group of normal synovial cells was not available. Moreover, OA is a disease that affects the entire joint. Therefore, further investigation is required to ascertain the effects of CeNPs on other tissues, such as chondrocytes and immune cells. Furthermore, metal-based formulations are challenging to modify and represent a significant impediment to targeted delivery. It is therefore recommended that the potential of employing pH-responsive, thermo-responsive, and photo-responsive properties as targeting modalities using hydrogel- and microneedle-based dosing methods be investigated.

Resveratrol-encapsulated gold nanoparticles (RGNPs) [11] are gold nanoparticle systems encapsulating resveratrol, an antioxidant polyphenol, as an anti-aging agent to delay cataracts. The mechanism of action of RGNPs is the activation of the Sirt1/Nrf2 pathway in the body, which significantly reduces oxidative damage (Figure 8). This includes a reduction in ROS generation, GSH consumption, and H_2_O_2_-induced MDA generation. Additionally, RGNPs have been observed to reduce aging, which in turn reduces SASP secretion and delays cataract development. The efficacy of RGNPs in vitro was demonstrated as follows: RGNPs were observed to significantly reduce oxidative damage, including ROS generation, intracellular glutathione (GSH) consumption, and H_2_O_2_-induced malondialdehyde (MDA) generation. Additionally, they were demonstrated to reduce the percentage of SA-β-gal-positive cells and the expression of senescence markers (p16, p21, BAX, BCL-2, and SASP) and cellular senescence to a greater extent than resveratrol. In terms of physicochemical properties, the charge was −25.27 mV, the average particle size was 35 nm (TEM), and the average particle size distribution was 78.8 nm. As also shown in Figure 1, DNA damage caused by aging stress or physiological processes activates p53, which in turn activates the CDK2 inhibitor p21^WAF1/Cip1^ (CDKN1A). The CDK4/6 inhibitor p16INK4A (CDKN2A) is also activated by DNA damage. These factors dephosphorylate and persistently activate RB family proteins, resulting in inhibition of E2F transcriptional activation, cell cycle arrest, and lifelong accumulation of senescent cells. RGNP activates the Sirt1/Nirt2 pathway, thereby lowering the abovementioned p21 and p16, GSH, ROS, and MDA and suppressing cellular senescence, as well as SA-β-Gal and SASP, thereby exerting anti-aging and anti-inflammatory effects.

The advantage of RGNPs is that, in vivo, RGNPs have been shown to activate the Sirt1/Nrf2 pathway in the body, inhibiting lens aging and delaying the onset of cataract development. After the administration of RGNPs to cataract model mice, the eyes were removed and proteins were extracted for Western blotting. Aging markers (p16 and p21) were significantly elevated in the cataract model group but decreased after RGNP treatment compared to after resveratrol treatment. The in vitro experimental results are in agreement with the results of the in vitro experiments: protein expression levels of Sirt1 and Nrf2 were significantly decreased in the cataract group and significantly increased in the RGNP group compared to the resveratrol group. On the other hand, a limitation of RGNPs is that, like CeNPs, they are metal-based formulations that are difficult to modify and target. The potential for targeting may be achieved through the utilization of gold colloid-labelled antibodies as a method of modification. Although they act on the eye, it is possible that they may migrate to other tissues, and therefore pharmacokinetic evaluation and toxicity assessment would need to be performed.

The following validations and issues may be considered to ensure the clinical safety and validity of RGNP formulations for clinical application. It should be noted that an in vivo toxicity evaluation was not conducted in this study, despite the completion of an in vitro evaluation. Given that the substance is a metal and that no targeting of specific tissues was conducted, there is a possibility of distribution to other tissues and effects on immunity. Accordingly, in vivo toxicity and pharmacokinetic evaluation should be conducted prior to clinical application.

## 7. Discussion

Analysis of the molecular biology of age-related diseases has not only helped determine disease mechanisms and therapeutic targets but has also led to the development of DDSs that can aid in developing more efficient treatment strategies. There is great potential for the future development of DDSs targeting age-related diseases and the development of formulations that have high therapeutic efficacy while reducing adverse reactions. In addition to methods to eliminate senescent cells, non-viral vectors suitable for genetic modification of senescent cells using niosomes have also been developed, and methods such as rejuvenating senescent cells are being investigated [34]. Furthermore, DDS technologies have been developed not just to target senescent cells but also to treat other age-related diseases. An example in oncology is therapy using senescent cell-destroying CAR-T cells that induce cancer cell senescence and recognize factors on the surface of senescent cells. Another is inducing apoptosis in senescent cancer cells using senolytics, which are drugs that recruit immune cells that induce and enhance SASP [26,35].

Although DDS formulations against senescent cells, as previously described, offer a number of advantages, they may also have limitations in terms of potential side effects and toxicity. The following section presents an overview of the potential adverse effects and toxicity associated with the formulations discussed in this review. No specific side effects of SA-β-Gal NPs have been documented in the literature. Information on the toxicity of SA-β-Gal NPs has been documented. It has been demonstrated that the encapsulation of nabitoclax in nanoparticles reduces the incidence of thrombocytopenia. Furthermore, the complete suppression of cardiac hypertrophy has been demonstrated to result in a reduction of adverse effects when doxorubicin is encapsulated. It has been demonstrated that drug encapsulation can result in a reduction in the incidence of adverse effects. A review of the literature reveals no specific side effects associated with sphingomyelin nanosystems. With regard to the toxicity of the sphingomyelin nanosystem, C18-PEG-Ks was demonstrated to exhibit cytotoxic effects in a dose-dependent manner, with an effective concentration of 5 µM. However, by binding to the nanoparticles, the effective concentration was achieved at a peptide dose of 0.5 µM, thereby effectively reducing the dosage. No direct adverse effects of si*FOXO4*-nanotubes (si*FOXO4*-NTs) have been reported in the literature. Toxicity information for si*FOXO4*-NTs has confirmed that removal of aged pulmonary endothelial cells by intraperitoneal injection or orally with FOXO4-DRI can worsen pulmonary hemodynamics in mouse models of pulmonary hypertension. The toxicity of FOXO4-DRI has been confirmed in a mouse model of pulmonary hypertension. Additional toxicological data indicate IC_50_ of 1457 nM for DNA nanotubes (DNA-NTs) and 157 nM for si*FOXO4*-NTs. The administration of magnetic EV-based i*Bax* mRNA/BTSA1 has been associated with a number of adverse effects, including minor pulmonary and splenic damage resulting from the accumulation of *iBax* mRNA. The likelihood of hepatotoxicity was reduced by the use of a modified *Bax* mRNA with a *miR-122-5p* recognition site in the 3′-UTR region, as was reported to be the case with this formulation. A review of the literature reveals no reports of adverse reactions associated with galactose-modified duocarmycin (GMD) prodrugs. In examining the toxicity of GMD, it was observed that when normal cells and senescent cells were co-cultured and exposed to GMD, low concentrations of 0 to 2.5 µM were found to kill some senescent cells without affecting normal cells. However, high concentrations of 5 µM were also observed to kill some normal cells. A thrombocytopenia side effect has been documented in association with Navitoclax. The administration of the Nav-Gal prodrug at the therapeutic dose for 10 days resulted in the absence of thrombocytopenia in the Nav-Gal treatment group, with platelet counts comparable to those observed in the control treatment group. This finding suggests a reduction in platelet toxicity relative to Navitoclax. Previous studies have reported the efficacy of Nav-Gal prodrugs in mitigating Navitoclax-induced thrombocytopenia at therapeutic doses. No specific adverse effects associated with TSPD have been documented in the scientific literature. Toxicity studies indicate that TSPD does not exhibit any discernible toxicity even at doses as high as 25 mg/kg over a two-week period. No specific side effects related to CeNPs have been documented in the scientific literature. With regard to the toxicity of CeNPs, no effect on cell viability has been observed at 100 μg/mL exposure, whereas a decrease in cell viability has been reported at 200 μg/mL. With regard to the toxicity of RGNPs, the CCK8 assay demonstrated that with increasing concentrations (2, 4, 8, 16, and 32 μg/mL) and incubation times (24 and 48 h), RGNPs resulted in minimal or no cell damage.

In addition to the aforementioned benefits of DDS technology for aging cells, it is possible to influence cellular metabolism and modify genetic material, but challenges remain. This is particularly the case with regard to nucleic acid DDSs. For instance, the i*Bax* mRNA/BTSA1 delivery EV^iTx^ [9] was reported to utilize a modified *Bax* mRNA comprising a *miR-122-5p (miR-122)* recognition site within the 3′-UTR region. This approach was taken in order to prevent hepatotoxicity and minimize side effects by avoiding hepatic metabolism. Furthermore, this method has been demonstrated to reduce the incidence of adverse effects by circumventing hepatic metabolism. Furthermore, this method circumvents translation in the liver through genetic modification. In terms of drug efficacy, *Bax* mRNA and the activator BTSA1 are delivered directly to senescent cells simultaneously, where they are activated by the activator to promote programmed elimination and kill senescent cells. Additionally, si*FOXO4*-NTs [5] have been demonstrated to be efficacious in tobacco smoke extract (CSE)-induced senescent lung fibroblasts. *FOXO4* siRNA was delivered to the lungs for FOXO4 knockdown in CSE-induced senescent HFL-1 cells, resulting in a concentration- and time-dependent reduction in FOXO4 levels in CSE-induced senescent HFL-1 cells. Gemcitabine, which is bound to TSPD, is an anti-metabolite. Following intracellular metabolism, it exerts its anti-tumor effect by inhibiting DNA synthesis. As previously outlined, the delivery of mRNA and siRNA can influence gene modification and metabolic processes. Conversely, metabolism and genes may also be affected as a consequence of the administration of drugs with anti-cancer properties.

 In consideration of the previously mentioned factors, it can be argued that, of the DDSs under review, EV^iTx^ [9], which can be employed to deliver i*Bax* mRNA/BTSA1, has the most extensive range of potential applications. This conclusion is based on four factors: (1) the variety of diseases for which it can be applied; (2) its targeting ability; (3) its side effects and toxicity; and (4) the stability, biocompatibility, and immunogenicity of the formulation. (1) The initial topic of discussion was the extensive range of disease categories that can be targeted. The objective of i*Bax* mRNA/BTSA1 delivery EV^iTx^ therapy is to eliminate senescent cells. This is achieved by simultaneously delivering *Bax* mRNA and the activator BTSA1 directly to the cells in question. This process is known as “activating the activator” and is designed to promote programmed elimination. This indicates that the mRNA can be utilized not only in the treatment of atherosclerosis but also in the management of other diseases where senescent cells are accumulated, contingent on the targeting. Moreover, although Bax protein was expressed in this study, other mRNAs, such as *FOXO4* [5], can be selected for use against a wider range of diseases. (2) The targeting of this formulation employs superparamagnetic iron oxide nanoparticles (SMNs) to achieve endovascular delivery, which is dependent on spatial recognition and independent of the type of cells and modification of the particles. This allows for the delivery of the drug to various types of cancer. It is believed that the drug delivery can be freely moved according to the location of the disease, and thus that it can be applied to various diseases. (3) With regard to the potential for adverse effects and toxicity, this preparation has been demonstrated to accumulate in the liver and exhibit hepatotoxicity. To circumvent this issue, a modified *Bax* mRNA with a *miR-122-5p (miR-122)* recognition site in the 3′-UTR region is employed to preclude translation between the two and thereby minimize the incidence of adverse effects. The aforementioned methodology allows for the avoidance of unintended side effects by incorporating a translation-avoiding modification into the 3′-UTR region, thus preventing the accumulation or localization of the compound in other tissues. (4) With regard to the stability, biocompatibility, and low immunogenicity of the formulation, this formulation is highly biocompatible for nucleic acid delivery due to the use of EV as a carrier, and it is also safe and stable as a result of its low immunogenicity. In consideration of these four points, it can be concluded that the EV^iTx^ delivery system, based on i*Bax* mRNA/BTSA1, exhibits the broadest range of potential applications within the context of this review.

In conclusion, we have examined the prospective applications of senescent cell targeting technology. Emerging DDS technologies include extracellular vesicles (EVs), including exosomes, microbubbles, metal–organic frameworks (MOFs) [36], hybrid polymer–lipid nanoparticles [32], and hydrogel formulations. In addition, materials have been developed that further improve targeting by adding features such as DDS technology using 3D printing technology [37], pH sensitivity [38], magnetism [9], and light sensitivity [39].

A novel approach to DDS research has emerged in recent years, namely, the introduction of proteins into cells [40]. By linking a series of sulfonic acids and introducing carbonates, modifiers have been developed. A modifier changes the surface charge of a protein and allows direct delivery of the protein by encapsulation in cationic liposomes. Due to their molecular weight and hydrophobicity, proteins have been difficult to deliver directly into cells. Many nucleic acid-based drugs are currently in research and development, as discussed in this review. However, due to their high immunogenicity and off-target effects, there are safety concerns regarding nucleic acid-based drugs. Protein-based drugs overcome the concerns around nucleic acid-based drugs and have proven to be highly effective in the suppression of various diseases. This technology can be applied to senescent cells, and various applications are possible, including gene editing of senescent cells and destruction of senescent cells using antibodies.

As candidates for new senescent cell markers, transcriptomic and proteomic analyses have identified RNAs and proteins that are increased or decreased compared to non-aging cells [17]. Profiles of RNAs expressed in eight experimental models of cellular senescence commonly studied using WI-38 and IMR-90 fibroblasts, HUVECs, and HAECs have been reported [17,41]. This study identified 50 upregulated and 18 downregulated transcripts that are commonly upregulated in all comparison pairs and can be used as novel biomarkers for senescent cells [41]. Fifty upregulated transcripts were identified, including lncRNA *PURPL* (p53 upregulator), SRPX, STAT1, TMEM159, and CCND3 [41]. On the other hand, 18 downregulated transcripts were identified, including HIST1H1D, HIST1H1A, HIST1H1E, HIST2H2AB, MCUB, FAM129A, and ANP32B, which encode histones [41]. Biomarkers that are expressed on the surface of senescent cells are very attractive targets for DDSs. Biomarkers on the surface of senescent cells that have been developed and used to target DDSs include CD9 [42], TSP-1/CD47 [4], and ApoD [8]. In addition, proteomic analysis has shown that DPP4 is strongly upregulated on the plasma membrane of senescent cells, allowing its separation by flow cytometry [17,43]. Besides DPP4 [17,43], other novel biomarkers that appear on aging cells and could be used as targets for DDSs include secreted carrier membrane protein 4 (SCAMP4) [44], DEP1 [45], B2MG [45,46,47], CD264 [48], CD36 [49], ICAM-1 [50], MDA-vimentin [51], NOTCH1 [52] and NOTCH3 [53], MIC A/B and ULBP2 [54,55,56], and uPAR (urokinase plasminogen activator) [57]. A review on novel biomarkers by Rossi M. published in 2021 [17] provides further details on this topic. Attractive targets may include novel biomarkers such as those mentioned above.

## 8. Summary

**Figure 9:**
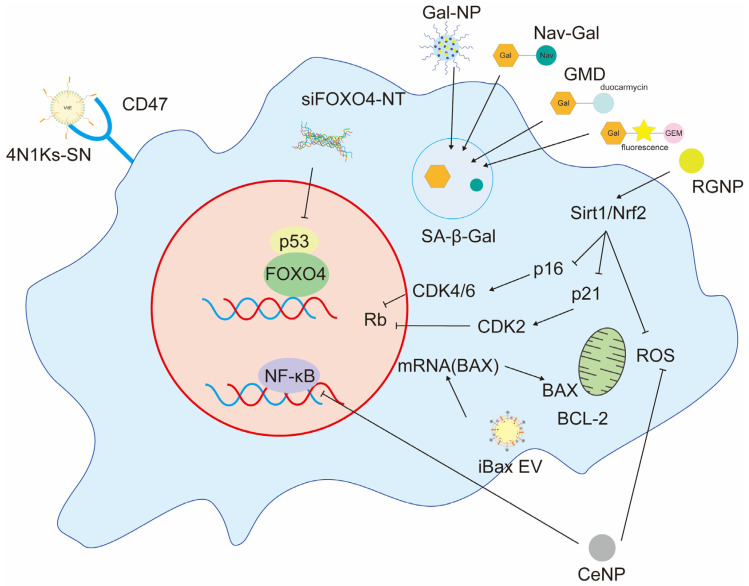
A schematic representation of the major senescent cell-targeted DDSs that have been published within the last five years.

DDSs for age-related diseases are becoming more diverse and numerous (Figure 9), and the field holds great promise for the development of next-generation DDSs. As described above, senescent cells can disrupt tissue homeostasis and contribute to inflammation-induced pathologies [58]. In fact, removing senescent cells has been shown to ameliorate signs of aging in mice, extending the average lifespan of mice by 24–27% [25]. The elimination of senescent cells may be detrimental to the homeostasis and health of mammals, as some studies in mice have recently reported [59]. In some specific organs and tissues, it is beneficial to eliminate certain types of senescent cells. However, the removal of too many other types of senescent cells can have detrimental effects [58]. Therefore, senolytic DDS aging therapy can be a promising way to selectively eliminate pathological senescent cells while sparing healthy senescent cells.

## Figures and Tables

**Table 1:** Major DDSs related to age-related diseases reported in the past 5 years. Indicate by ○ if an experiment for drug evaluation is being conducted, ― if not, and △ if experiments are being conducted but not for drug evaluation.

Classification	DDS	Target	Drug/Active Ingredient	Target Cells/Target Diseases	Reference	In Vitro	In Vivo
Sphingomyelin nanosystem	4N1Ks- sphingomyelin nanosystem	TSP-1/CD47 receptor	4N1K peptide	Senescent cells/breast and colon cancer	[4]	○	―
DNA self-assembled nanoparticles(NPs)	*FOXO4*siRNA-loaded DNA self-assembled nanoparticles	FOXO4	FOXO4siRNA	Senescent cells/Chronic Obstructive Pulmonary Disease (COPD)	[5]	○	△ (Genetic analysis only)
Prodrug	Galactosidase (Gal)-modified duocarmycin prodrug (GMD)	β-galactosidase	Duocarmycin	Senescent cells (adamantinomatous craniopharyngioma (*Hesx1^Cre/+^*; *Ctnnb1^lox(en3)/+^*ACP mouse model), β-catenin-positive precancerous aged cells)	[6]	○	○
Prodrug	Gal-bound Navitoclax (Nav-Gal)	β-galactosidase	Navitoclax	Senescent cell/human A549 lung cancer cell subcutaneous tumor xenograft SCID mice, KP lung adenocarcinoma cell orthotopic transplantation model mice	[7]	○	○
Prodrug	Prodrug TSPD for chronic renal failure	β-galactosidase	Gemcitabine	Chronic renal failure	[8]	○	○
Magnetic+EV+nucleic acid	Magnetic EV-based i*Bax*mRNA/BTSA1 delivery	BCL-2 related protein	i*Bax*mRNA/BTSA1	Atherosclerosis (AS) (ApoE^−/−^ mouse)	[9]	○	○
CeNPs	Ceria nanoparticles (CeNPs)	ROS/NF-κB	Ceria nanoparticles	Osteoarthritis	[10]	○	○
Gold nanoparticles	Resveratrol-encapsulated gold nanoparticles (RGNPs)	Sirt1/Nrf2	Resveratrol	Cataracts	[11]	○	○
